# Neoadjuvant Systemic Therapy in Early Breast Cancer: Results of a Prospective Observational Multicenter BRIDE Study

**DOI:** 10.3390/cancers15194852

**Published:** 2023-10-04

**Authors:** Stefania Gori, Alessandra Fabi, Catia Angiolini, Monica Turazza, Piermario Salvini, Gianluigi Ferretti, Elisabetta Cretella, Lorenzo Gianni, Claudia Bighin, Angela Toss, Claudio Zamagni, Patrizia Vici, Costanza De Rossi, Antonio Russo, Giancarlo Bisagni, Antonio Frassoldati, Lucia Borgato, Anna Cariello, Claudia Cappelletti, Roberto Bordonaro, Saverio Cinieri, Alessandra Modena, Matteo Valerio, Maria Francesca Alvisi, Irene De Simone, Francesca Galli, Eliana Rulli, Anna Santoni, Fabrizio Nicolis

**Affiliations:** 1 Medical Oncology Unit, IRCCS Sacro Cuore Don Calabria, 37024 Negrar di Valpolicella, Italy; 2 Precision Medicine Unit in Senology, Fondazione Policlinico Universitario A. Gemelli IRCCS, 00168 Rome, Italy; 3 Breast Unit and Multidisciplinary Oncology Group, Department of Breast Oncology, AOU Careggi, 50134 Florence, Italy; angiolinic@aou-careggi.toscana.it; 4 Oncology, Humanitas Gavazzeni, 24125 Bergamo, Italy; piermario.salvini@grupposandonato.it; 5 Oncological Medicine—Policlinico Ponte S Pietro di Istituti Ospedalieri Bergamaschi, 24036 Ponte San Pietro, Italy; 6 Division of Medical Oncology 1, IRCCS Regina Elena National Cancer Institute, 00144 Rome, Italy; 7 Oncology—Azienda Sanitaria dell’Alto Adige, 39100 Bolzano, Italy; 8 Oncology—AUSL Romagna Rimini, 47900 Rimini, Italy; 9 Oncology—IRCCS AOU San Martino IST, 16132 Genova, Italy; 10 Department of Oncology and Hematology, Azienda Ospedaliero-Universitaria di Modena, 41125 Modena, Italy; 11 Department of Medical and Surgical Sciences, University of Modena and Reggio Emilia, 41125 Modena, Italy; 12 Medical Oncology of Senology and Gynecology, IRCCS AOU Bologna, Policlinico Sant’Orsola, 40138 Bologna, Italy; 13 UOSD Sperimentazioni Fase IV, IRCCS Istituto Nazionale Tumori Regina Elena, 00144 Rome, Italy; 14 Oncology—Ospedale dell’Angelo Azienda ULSS 3 Serenissima, 30174 Venice, Italy; costanza.derossi@aulss3.veneto.it; 15 Medical Oncology, AOU Policlinico P. Giaccone, 90127 Palermo, Italy; 16 Medical Oncology, Comprehensive Cancer Centre, AUSL-IRCCS di Reggio Emilia, 22100 Reggio Emilia, Italy; giancarlo.bisagni@ausl.re.it; 17 Clinical Oncology, Sant’Anna University Hospital, 44124 Ferrara, Italy; 18 Department of Oncology, San Bortolo General Hospital, Azienda ULSS8 Berica, 36100 Vicenza, Italy; 19 Medical Oncology, AUSL Ravenna, 48100 Ravenna, Italy; anna.cariello@auslromagna.it; 20 Medical Oncology, Fano Hospital, 61032 Fano, Italy; 21 Medical Oncology, ARNAS Garibaldi Hospital, 95124 Catania, Italy; 22 Medical Oncology, Antonio Perrino Hospital, 72100 Brindisi, Italy; 23 Laboratory of Methodology for Clinical Research, Department of Clinical Oncology, Istituto di Ricerche Farmacologiche Mario Negri IRCCS, Via Mario Negri 2, 20156 Milan, Italyirene.desimone@marionegri.it (I.D.S.); eliana.rulli@marionegri.it (E.R.);; 24 Medical Direction, IRCCS Sacro Cuore Don Calabria, 37024 Negrar di Valpolicella, Italy

**Keywords:** early breast cancer, criteria of choice of neoadjuvant therapy, types of neoadjuvant therapy, pathological response

## Abstract

**Simple Summary:**

To assess the percentage of early breast cancer (EBC) patients treated with neoadjuvant systemic therapy (NAT) in Italy, criteria of patient selection and types of therapies delivered, 1276 stage I-II-III patients were enrolled and evaluated in the multicenter prospective observational BRIDE study in 2018–2021. NAT was administered to 13.9% of EBC patients. In multivariate analysis, menopausal status, cT, cN, grade, HER2-positive and Triple Negative (TN) subgroups were significantly associated with the decision to administer NAT. According to phenotypic subgroup, NAT was administered to 53.2% of HER2+/HR-negative (pathologic complete response—pCR-74.2%), 27.9% of HER2+/HR+ (pCR 52.3%), 7.1% of HER2-negative/HR+ (pCR 17.2%) and 30.3% of TN (pCR 37.9%) patients. Phenotypic subgroup influenced the type of NAT delivered. Today, the use of NAT in EBC should be always considered, especially in HER2+ and TN, because of the association between pCR and better survival of patients and the current availability of effective therapies for patients with residual disease.

**Abstract:**

To evaluate the rate of early breast cancer (EBC) patients treated with neoadjuvant systemic therapy (NAT) in Italy, criteria of patient selection and types of therapies delivered, an analysis of 1276 patients with stage I-II-III was conducted out of 1633 patients enrolled in the multicenter prospective observational BRIDE study. A total of 177 patients (13.9%) were treated with NAT and 1099 (85.9%) with surgery; in multivariate analysis, menopausal status, cT, cN, grade, HER2-positive and Triple negative (TN) subgroups were significantly associated with the decision to administer NAT. The type of NAT delivered was influenced by EBC subtype. NAT was administered to 53.2% of HER2+/HR-negative, 27.9% of HER2+/HR+, 7.1% of HER2-negative/HR+ and 30.3% of TN EBC patients. The pCR rates were similar to the ones reported in the literature: 74.2% in HER2+/HR-negative, 52.3% in HER2+/HR+, 17.2% in HER2-negative/HR+ and 37.9% in TN. In clinical practice, patient and tumor characteristics influenced oncologists in the decision to administer NAT in EBC and in the choice of the type of systemic therapy, according to ESMO and AIOM Guidelines. Currently, it is recommended always to evaluate the use of NAT in EBC, mainly in HER2+ and TN patients, considering that pCR is associated with significantly better survival of the patient and that effective therapies are now available for residual disease.

## 1. Introduction

Breast cancer is the most commonly diagnosed cancer in women (24.5% of 9.2 million new cases diagnosed in 2020) and the leading cause of cancer death in females (15.5% of 4.4 million deaths for cancer [1].

Also, in Italy, breast cancer represents the first cause of oncological death (12,300 deaths in 2017) [2] and the most frequently diagnosed neoplasm in women (55,000 new estimated cases in 2020) [3].

Due to the high incidence and good prognosis (5-year survival is 87%), its prevalence is also high: in Italy, 834,000 women with a previous diagnosis of breast cancer were alive in 2020 [3].

The majority (90–95%) of breast cancers are diagnosed at an early stage and only 5–10% of new breast cancers are metastatic de novo. The integration of the various therapeutic modalities (surgery, radiotherapy, systemic treatment, supportive therapies) and the collaboration of different specialists within multidisciplinary teams are fundamental to ensure the best treatment for each patient and achieve the best outcome [4,5].

In early breast cancer (stage I-II-III) patients, the knowledge of the clinical and biological prognostic factors [Tumor size, lymph node status, Grading, Estrogen Receptor (ER) and Progesterone Receptor (PgR) status, proliferative index (Ki67 value) and HER2 status] is very important in order to identify the most appropriate treatment options and decide about the start of NAT or refer the patient directly to the surgeon considering later an adjuvant therapy.

Today, neoadjuvant treatment is recommended for patients with locally advanced breast cancer, and for patients with resectable breast cancer. In locally advanced breast cancer patients, NAT is administered with the aim of performing surgery on patients who are not candidates for surgery yet. In patients with resectable disease, NAT is administered to reduce the size of the primary tumor, allowing conservative surgery, to achieve downstaging of axillary surgery (with consequently lower morbidity), to provide prognostic information based on pathological response, to allow the evaluation of the efficacy of neoadjuvant treatment with the possibility of progressive disease and to administer alternative, effective treatments in patients with residual disease.

To assess the rate of patients with early breast cancer treated with NAT, to understand the criteria for selecting patients for NAT and to describe the types of therapies administered, an analysis was performed in stage I-II-III patients with breast cancer enrolled in the BRIDE study.

## 2. Material and Methods

The BRIDE study was an observational, prospective, multicenter study.

The objectives of the BRIDE study were: to evaluate the distribution of patients with stage I-II-III breast cancer who were candidates to either systemic NAT or upfront surgery (followed by adjuvant therapy), and to determine both the parameters guiding the choice of systemic NAT and the type of NAT; to evaluate the types of treatment administered in stage IV patients at diagnosis (metastatic de novo) and in stage IV for recurrence of previous breast cancer. Secondary objectives were to estimate the disease-free survival (DFS), progression-free survival (PFS), overall survival (OS), and to compare the practice observed in Italian clinical settings with the practice proposed by AIOM v. 2017 Breast Cancer Guidelines [6].

Inclusion criteria were the following: female sex; 18 years old or older at time of diagnosis; histological diagnosis of in situ (DCIS, LCIS) or invasive breast carcinoma; stage 0-I-II-III-IV patient (according to TNM v. VII) [7]; availability of clinical and/or pathological parameters: Tumor (T), Node (N), Metastasis (M); availability of biological parameters: Grading, ER and PgR status, Ki67 value, HER2 status on primary tumor and/or metastatic lesion. According to the International Conference on Harmonization/Good Clinical Practice [ICH/GCP], patients must have signed the written informed consent before enrolment. No exclusion criteria were set for the BRIDE study.

Breast cancer was considered ER negative if <1% or 0% of tumor cell nuclei were immunoreactive. A similar principle was applied to PgR testing [8,9].

HER2 status was considered negative if equal to 0 or 1+ by immunohistochemistry (IHC) or if 2+ by IHC and not amplified (FISH/SISH/CISH). HER2 status was considered positive if 3+ by IHC or 2+ by ICH and amplified (FISH/SISH/CISH) or if amplified (FISH/SISH/CISH) [10].

The patients were classified in four phenotypic subgroups, as suggested by Cortazar [11], and based on HER2, ER and PgR values:

(a) HER2-positive/HR-positive (HER2+/HR+) subgroup included the patients with HER2-positive and ER and/or PgR positive breast cancer cells;
(b) HER2-positive/HR-negative (HER2+/HR-negative) subgroup included the patients with HER2-positive and ER and PgR negative breast cancer cells;
(c) HER2-negative/HR-positive (HER2-negative/HR+) subgroup included the patients with HER2-negative and ER and/or PgR positive breast cancer cells;
(d) Triple negative (TN) subgroup included the patients with HER2-negative and ER and PgR negative breast cancer cells.

The pCR was defined as absence of invasive disease cells in both breast and lymph nodes at surgery after NAT (ypT0 ypN0 or ypT0/is ypN0) [11].

The protocol was reviewed by the independent ethics committee of the coordinating center and by the ethics committees of each participating center [12]. The protocol complied with the recommendations of the 18th World Health Congress (Helsinki, 1964) [13].

### 2.1. Sample Size Determination

No formal statistical hypothesis for comparison was planned. It was estimated that 150 to 300 patients per center, per year would be available. According to the guidelines’ compliance objective, an agreement not lower than 80% approximately was expected. Assuming 50% to 100% variability in prevalence in each subgroup of patient populations (stage 0-I-II-III, IV), the precision of the statistical estimates (defined by the width of confidence interval of 95%) was calculated to vary between 3% and 5%.

According to these considerations, at least 4500 patients’ data had to be obtained. Because of a low accrual rate observed mainly due to the COVID-19 pandemic, enrollment was stopped prematurely, and the planned sample size was not reached.

### 2.2. Data Collection and Evaluated Variables

The source of data was patients’ medical records. Center characteristics, demographic and clinical characteristics of the patients, tumor characteristics, biological characterization of the primary tumor, and information regarding the treatments (adjuvant, neoadjuvant and metastatic settings) were collected as pseudonymized data and analyzed.

### 2.3. Statistical Methods

Patients’ characteristics were described through descriptive analysis. Continuous variables were described by the median, the first and third quartiles and minimum and maximum values (range). Categorical variables were described using the frequency and percentage of patients in each category. Univariable and multivariable logistic regression models were performed statistically to detect and estimate clinical-pathological differences between patients treated with NAT and patients referred to upfront surgery. Results of the analysis were expressed as odds ratio (ORs), 95% confidence intervals (95% CIs) and *p*-values.

The associations between the treatment choice (NAT or upfront surgery) and the phenotypic subgroup were assessed by means of Chi-squared tests. The Fisher test was performed to evaluate the association between the type of NAT and the phenotypic subgroup. The *p*-values < 0.05 were considered statistically significant. Analysis was carried out using the SAS (Statistical Analysis System, SAS Institute, Version 9.4, Cary, NC, USA) software.

## 3. Results

**Figure 1:**
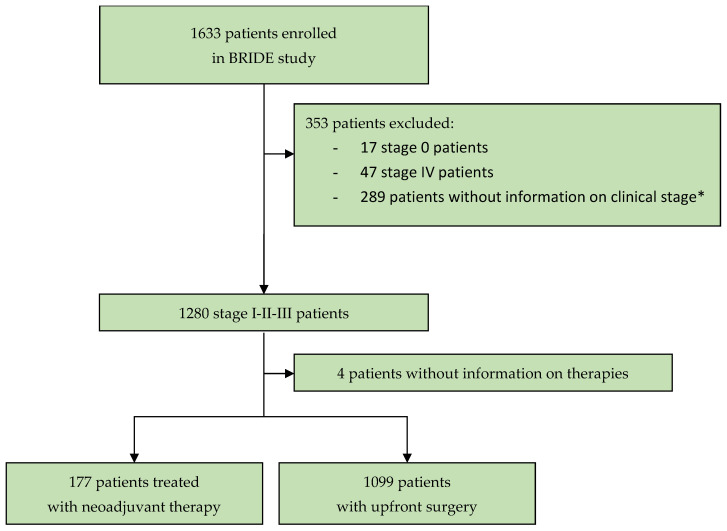
Study profile. * In this analysis, 289 patients were not evaluated because information on clinical stage at diagnosis was needed to assess the criteria used by oncologists for starting neoadjuvant systemic therapy.

From 8 January 2018 to 3 February 2021, 1633 patients with diagnosed breast cancer were enrolled in the BRIDE study from 19 Italian cancer centers. To assess the distribution of patients with stage I-II-III breast cancer to the groups “candidate to systemic NAT” or “upfront surgery”, and to determine the parameters to assign patients to systemic NAT and the type of NAT, this analysis evaluated 1276 patients at stage I-II-III with information on their clinical stage at diagnosis (Figure 1). The data snapshot for this analysis was carried out on 26 May 2023.

The median follow-up is 32.6 months (interquartile range: 3.0 to 44.1 months).

### 3.1. Neoadjuvant Therapy and Oncologists’ Choice

Out of the 1276 stage I-II-III patients, 177 (13.9%; 95% CI 12.1–15.8%) were treated with NAT and 1099 (86.1%; 95% CI 84.1–88%) with upfront surgery followed by adjuvant therapy (Table 1).

Clinical and tumor characteristic differences between NAT patients and upfront surgery patients were evaluated by univariable logistic models and summarized in Table 1. Statistically significant associations were found with the use of NAT in terms of age (median age 50.4 years in NAT patients vs. 62.4 years in surgical patients; *p* < 0.001), premenopausal status (54.5% vs. 28.7%; *p* < 0.001), clinical tumor size > 2 cm (87% vs. 24.8%; *p* < 0.001), positive clinical nodal status (55.9% vs. 21.4%; *p* < 0.001), grading G3 (61.7% vs. 25.2%; *p* < 0.001), Ki-67 value ≥ 20% (62.8% vs. 40.3%; *p* < 0.001). Lastly, a higher percentage of HER2-positive and triple negative subgroups were observed in patients treated with NAT.

Multivariable analysis was performed on 1070 patients with no missing data to evaluate the association between the treatment choice and the variables that had resulted statistically significant at univariate analysis; it showed that menopausal status, cT, cN, grade, HER2-positive and Triple negative subgroups were still significantly associated with the decision to administer NAT (Table 2).

To evaluate the role of the phenotypic subgroup in the oncologists’ choice to administer NAT, the proportion of patients treated with NAT in each subtype was reported in Table 3. NAT was administered to 33 (53.2%) HER2+/HR-negative patients, to 46 (27.9%) HER2+/HR+ patients, to 66 (7.1%) HER2-negative/HR-positive patients and to 30 (30.3%) triple negative breast cancer patients.

Table 4 describes the oncologists’ choice to administer NAT in patients with cT > 2 cm and/or lymph node positivity (N+). Out of 556 patients with cT > 2 cm and/or lymph node positivity (by clinical/imaging and cytology), 160 (28.8%; 95% CI 25–32.5%) received NAT. More specifically, NAT was delivered to 42 (44.2%) HER2+/HR+ patients, to 29 (69.1%) HER2+/HR-negative patients, to 63 (17.2%) HER2-negative/HR+ patients and to 26 (49.1%) triple negative breast cancer patients. In these patients, a statistically significant difference between the choice of administering NAT and the phenotypic subgroups was found (*p* < 0.0001).

### 3.2. Type of Neoadjuvant Systemic Therapy

The type of NAT administered differed according to the phenotypic tumor subgroup. As shown in Table 5, among the 177 patients treated with NAT, the type of NAT administered was associated with the phenotypic subgroup (Fisher *p*-value < 0.0001). Chemotherapy with the anti-HER2 agent was administered to 39 (84.8%) HER2+/HR+ patients and to 31 (93.9%) HER2+/HR-negative breast cancer patients. Chemotherapy alone was delivered to 59 (89.4%) HER2-negative/HR+ patients and to all 30 (100%) patients with a triple negative breast cancer.

Table 6 reports the neoadjuvant chemotherapy regimens administered (with or without anti-HER2 agent) according to breast cancer phenotypic subgroup. Anthracycline and taxane-based was the most frequently used chemotherapy regimen: for 33 (71.7%) HER2+/HR+ patients, 21 (63.6%) HER2+/HR-negative patients, 58 (98.3%) HER2-negative/HR+ patients and 28 (93.3%) triple negative breast cancer patients.

### 3.3. Surgery and Pathological Response

Among the 177 patients treated with NAT, 168 (94.9%) underwent breast surgery: mastectomy was performed in 95 (53.9%) patients and conservative surgery in 73 (41.5%) patients; 8 (4.5%) patients did not undergo breast surgery, and no information was collected for a patient lost to follow-up (Table 7).

Axillary surgery was performed in 166 (93.8%) of these 168 patients.

Only sentinel lymph node biopsy (SLNB) was performed in 87 out of 166 patients (52.4%) and only axillary lymph node dissection (ALND) in 53 (31.9%) patients. Axillary lymph node dissection after SLNB was conducted in 26 (resulted SLNB+) out of 166 patients (15.6%) (Table 7).

Out of 113 patients treated with SLNB, 26 (23.0%) patients resulted in lymph-node positive (N+) and underwent subsequent axillary lymph node dissection: 8 of these 26 (30.8%) patients had other positive lymph nodes.

Among all the 53 patients that underwent ALND only, 33 (62.2%) patients resulted node positive (N+), and 20 (37.7%) patients resulted node negative (N0).

About downstaging of the axilla after NAT, it was observed in 99 patients with cN-positivity at diagnosis that, after surgery, 30.7% of patients were pN0 and 6.8% had only micrometastases.

The pathological response observed in all 177 patients treated with NAT was reported in Table 8: pCR was observed in 68 (40.5%) patients, pathological partial response (pPR) in 88 (52.4%) patients, stable disease (SD) in 7 (4.2%) patients and Progressive Disease (PD) in 5 (3.0%) patients.

The proportion of pathological response was different according to the phenotypic subgroup.

pCR was reported in 23 (52.3%) HER2+/HR+ patients, in 23 (74.2%) HER2+/HR-negative patients, in 11 (17.2%) HER2-negative/HR+ patients and in 11 (37.9%) triple negative breast cancer patients.

pPR was reported in 20 (45.5%) HER2+/HR+ patients, in 7 (22.6%) HER2+/HR-negative patients, in 48 (75.8%) HER2-negative/HR+ patients and in 13 (44.8%) triple negative breast cancer patients.

Overall, PD was observed only in 5 out of 177 patients (3.0%). In detail, in 1 patient with HER2-/HR+ tumor (treated with chemotherapy only; cT2/cN1) and in 4 patients with triple negative tumors, all treated with chemotherapy only (1 patient cT2/cN0, 1 patient cT2/cN3, 1 patient cT3/cN1 and 1 patient cT4/cN0).

### 3.4. Adjuvant Treatment

After NAT and surgery, adjuvant systemic therapy was administered to 144 out of 177 patients (81.4%).

Table 9 shows the patients treated with adjuvant systemic therapy, with or without anti-HER2 agent(s), according to phenotypic subgroup and pathological response.

In the triple negative subtype, adjuvant chemotherapy was not administered to 11 patients with pCR after NAT, while it was administered to 12 out of 14 (85.7%) patients with residual disease.

In HER2+ patients (irrespective of the HR status), adjuvant therapy with anti-HER2 agent(s) was delivered to 40 out of 44 (90.9%) patients with pCR after NAT; and to 27 out of 28 (96.4%) patients with residual disease. Adjuvant TDM-1 was administered to only 7 out of these 28 (25%) HER2+ patients (irrespective of the HR status) with residual disease after neoadjuvant treatment.

Hormonal adjuvant therapy (alone or in combination with anti-HER2 agent) was administered to 84 out of 100 (84%) patients with hormonal receptor positive disease.

Adjuvant radiotherapy was delivered to 110 out of 168 patients (65.1%). Adjuvant radiotherapy was administered to 62 out of 73 (84.9%) patients who had undergone conservative surgery, and to 47 out of 95 (49.5%) patients who had undergone mastectomy.

## 4. Discussion

Between January 2018 and February 2021, 1633 patients with diagnosed breast cancer were enrolled from 19 Italian cancer centers to take part in the BRIDE study, an observational prospective multicenter study.

In order to evaluate the percentage of early breast cancer patients treated with neoadjuvant systemic therapy in Italy, the variables that determined the choice of administered NAT, and the types of systemic therapy delivered, an analysis was conducted of 1276 patients with early breast cancer (stage I-II-III) and information on clinical stage at diagnosis.

The results showed that 177 patients (13.9%) were treated with NAT and 1099 (86.1%) with upfront surgery (followed by adjuvant therapy).

After the multivariable analysis, menopausal status, cT, cN, grade, HER2-positive and triple negative subgroups were significantly associated with the decision to administer NAT (Table 2). Despite the overall low percentage of patients treated with NAT, a different rate has been observed in the various tumoral subtypes: actually, NAT was administered to 53.2% of HER2+/HR-negative patients, 27.9% of HER2+/HR+ patients, 7.1% of HER2-negative/HR-positive patients and 30.3% of triple negative breast cancer patients (Table 3). The number of patients treated with NAT was statistically associated to their respective phenotypic subgroup (*p* value < 0.0001).

We observed that, in clinical practice, cT > 2 cm and/or lymph node positivity (N+) influenced the oncologists’ choice to administer NAT: in fact, 28.8% (160/556) of patients with these characteristics received NAT in comparison to 13.9% reported in all 177 patients. In the presence of cT > 2 cm and/or N+, NAT was administered to 44.2% of HER2+/HR+ patients, 69.1% of HER2+/HR-negative patients, in 17.2% of HER2-negative/HR+ and 49.1% of triple negative breast cancer patients (*p* < 0.0001).

In the BRIDE study, the variables that influenced oncologists in their decision to start NAT are those reported in the European Society for Medical Oncology (ESMO) [14] and AIOM guidelines [6,15]: these results emphasized that most centers were fully in line with scientific guidelines.

Our results showed that the type of neoadjuvant systemic therapy was driven by tumor subtype: chemotherapy with anti-HER2 agent was delivered to 75.0% of HER2+/HR+ and to 93.9% of HER2+/HR-negative patients; only chemotherapy to 89.1% of HER2-negative/HR+ and to 100% of triple negative patients (Table 5).

The definition of pCR used in this study was ypT0/is ypN0, as suggested by the CTNTNeoBC pooled-analysis of 12 trials with 11,955 patients, which showed that the presence or absence of ductal carcinoma in situ did not affect long-term outcome [11].

Our results reported different pCR rates in the various tumor subtypes (52.3% in HER2+/HR+, 74.2% in HER2+/HR-negative, 17.2% in HER2-negative/HR+ and 37.9% in triple negative breast cancer), but these percentages resulted similar to or higher, for each subgroup, than what is reported in the literature [11,16].

Unfortunately, as we do not have information on the rate of patients treated with NAT in Italy prior to 2018, the year when enrollment in the BRIDE study began, we are unable to determine whether the rate observed during enrolment in the BRIDE study (2018 to 2021) has increased over time. However, today it is important for oncologists always to consider the use of NAT in patients with EBC, in light of the achievable outcomes reported in the literature.

The use of NAT and the importance of pCR have grown over the decades. In the late 1990s and early 2000s, two large randomized trials (NSABP B-18 and B-20) reported no difference in disease-free survival and overall survival between neoadjuvant and adjuvant therapy [17,18], but showed that patients with pCR had a significantly superior disease-free survival and overall survival compared with patients who did not [19,20,21]. In 2004, a CTNTNeoBC pooled-analysis [11] reported a positive association between pCR and long-term outcomes, both event-free survival (EFS) and overall survival (OS), in HER2+/HR-negative, HER2+/HR+, triple negative and HER2-negative/HR+ G3 tumors. This association was strongest in triple negative and in HER2+/HR-negative tumors treated with trastuzumab. Similar results were reported by a comprehensive meta-analysis of 52 studies with 27,895 patients [22]. Cortazar et al. reported that this positive association between pCR and survival was strong at the patient level but little at the trial level [11]. This observation has recently been confirmed in HER2+ EBC: pCR is strongly prognostic for EFS and OS in individual patients [23], but it is not associated with better EFS and OS in neoadjuvant trials of HER2+ operable breast cancer [24]. This evidence confirms the importance of considering NAT in EBC, especially in HER2+ and TN tumors: achieving pCR in the individual patient is associated with a better outcome than in patients with residual disease.

Today is used the definition of pCR (ypT0/is ypN0) suggested by Cortazar [11]. In this definition, the presence or absence of DCIS in residual disease did not affect long-term outcome. However, some evidence suggests that DCIS with HER2-overexpressed and HR-negative may be associated with the highest rates of ipsilateral breast cancer recurrence, and future studies are needed to evaluate the possible prognostic and predictive role of HER2 in DCIS, also in residual disease after NAT [25].

In our study, adjuvant systemic therapy was administered following NAT and surgery to 81.4% of patients, in compliance with ESMO [14] and AIOM Guidelines [6,15] and considering their phenotypic subgroup and pathological response. In patients with residual disease after NAT, adjuvant therapy with anti-HER2 agent(s) was administered to 96.4% (27/28) of HER2+ breast cancer patients (both HR+ and HR-negative). However, adjuvant TDM-1 was administered to only 25% of HER2+ patients with residual disease, because, during the enrolment period (from January 2018 to January 2021), the use of T-DM1 with this indication was available in Italy under a compassionate program from September 2019. AIFA approved TDM-1 in September 2021 (Official Gazette no. 232 of 28 September 2021) [26] in light of the results of the Katherine trial [27] which reported an invasive disease-free survival at 3 years of 88.3% in the T-DM1 group in comparison with 77.0% in the trastuzumab group (HR 0.50; 95% CI, 0.39 to 0.64; *p* < 0.001).

In triple negative breast cancer patients with residual disease, adjuvant chemotherapy was given in 85.7% of cases, according to the results of the Create-X study, which showed in triple negative patients with residual disease a 5-year DFS of 69.8% in the capecitabine group versus 56.1% in the control group (HR 0.58; 95% CI, 0.39 to 0.87) and an overall survival of 78.8% versus 70.3% (HR 0.52; 95% CI 0.30 to 0.90) [28].

In HR+/HER2-negative patients with residual disease, endocrine therapy was given in 90% (47/52) of cases.

However, for TN patients, HR+/HER2-negative patients and for HER2-negative patients with germline pathogenic or likely pathogenic variants in germline BRCA1 or BRCA2 (gBRAC1/2pv) with residual disease after NAT, more drugs are available today in Italy than they were during the BRIDE study enrolment period.

In triple negative patients with residual disease after neoadjuvant pembrolizumab and chemotherapy, adjuvant therapy is advised with pembrolizumab according to results of phase III trial KEYNOTE 522 [29]. In addition to reporting the improved rate of pCR and 3-year EFS in the pembrolizumab + chemotherapy arm compared with placebo + chemotherapy (EFS: 84.5% versus 76.8%; HR for event or death, 0.63; 95% CI, 0.48 to 0.82; *p* < 0.001), this trial showed a longer 3-year EFS among patients with residual disease treated with pembrolizumab versus placebo (67.4% versus 56.8%; HR for event or death: 0.70; 95% CI, 0.52 to 0.95) in the above-specified exploratory analysis [30,31].

In HR+/HER2-negative patients with residual disease after NAT, a CDK-4/6 inhibitor is now available in the adjuvant setting: it is abemaciclib for 2 years in combination with standard endocrine therapy, based on the results of the monarchE trial. This phase-3 study enrolled 5634 EBC patients at high risk after adjuvant chemotherapy (62%) or neoadjuvant chemotherapy (36%) and locoregional treatment (with ≥4 positive pathologic axillary lymph nodes (N+) or 1 to 3 N+ and at least one of the following: tumor size ≥ 5 cm or histologic grade 3 at surgery) randomized to abemaciclib and endocrine therapy versus endocrine therapy, reported a 4-year invasive-DFS of 85.8% (95% CI 84.2–87.3) in the abemaciclib plus endocrine therapy group vs. 79.4% (77.5–81.1) in the endocrine therapy alone group, with an absolute difference in invasive-DFS of 6.4% [32,33].

For triple negative and HR+/HER2-negative patients with gBRAC1/2pv and residual disease after NAT, today a PARP-inhibitor-Olaparib is available based on the results of the OlympiA trial, which showed that adjuvant olaparib administered for one year generates significantly longer invasive-DFS, distant-DFS and overall survival than placebo [34,35].

HR+/HER2-negative patients must have residual disease with a CPS + EG score of 3 or higher.

Our study has several potential limitations.

First, due to low accrual partly because of the COVID-19 pandemic, enrollment was stopped prematurely at 1633 patients, and then the planned sample size was not reached.

Second, methods used in pathology laboratories to assess residual disease were not collected. Today, there are many methods to evaluate the residual disease used in clinical practice: Residual Cancer Burden, CPS + EG score and Pinder.

Residual Burden Cancer (RBC), which was designed in 2007 to provide a standard method to evaluate and quantify the extent of residual disease in breast and axillary lymph nodes following neoadjuvant chemotherapy [36], includes four scores from RCB-0 (pCR) to RCB-3, that represent an increasing residual cancer burden. RCB assessments were highly reproducible among pathologists and both RCB and four classes have been validated as prognostic in many trials [37] and, recently, in different subtypes of early breast cancer with regard to EFS at 3, 5 and 10 years in pooled analysis of 5161 patients treated with neoadjuvant chemotherapy between 1994 and 2019 [38].

The CPS + EG score (Clinical-Pathologic scoring System) includes a pre-NAT clinical stage and a final pathological stage after NAT with incorporation of Estrogen receptor status and histologic Grade (EG) to provide prognostic information (overall survival, distant-metastasis-free survival, disease specific survival) in patients treated with NAT [39,40]. The CPS + EG score was used in the OLYMPIA trial to select high risk HR + EBC patients with gBRCAm and residual disease (CPS + EG score ≥ 3) [34,35].

Pinder evaluates the response to neoadjuvant chemotherapy using a five-point histological grading system, the key feature of which is the reduction in tumor cellularity; the comparison is made with a pre-treatment core biopsy. This classification system can predict overall survival and disease-free interval in patients treated with NAT [41].

Finally, in this first analysis performed at a median follow-up of 32.6 months, disease recurrence and survival analyses were not reported, since the observed follow-up of included patients was not long enough to perform a reliable and informative analysis. The analyses of these endpoints will be performed when the follow-up is mature, and their results will be reported in a following paper.

## 5. Conclusions

The results of the BRIDE study showed that, in clinical practice, patient and tumor characteristics reported in ESMO and AIOM Guidelines influenced oncologists in their choice of whether administering a neoadjuvant systemic therapy in early breast cancer. The rate of I-II-III stage patients treated with NAT was 13.9%, but this percentage is different in the various EBC subtypes. The pCR rates reported in clinical practice in phenotypic subgroups were similar to the ones reported in the literature.

However, it is today necessary for oncologists carefully to consider NAT in early breast cancer patients, mainly in HER2+ and in triple negative subtypes, according to ESMO and AIOM guidelines, because NAT also allows the assessment of response to therapy, which has an established prognostic value and may guide the choice of post-operative treatment.

## Figures and Tables

**Table 1:** Stage I-II-III breast cancer patients treated with neoadjuvant therapy vs. patients who underwent upfront surgery: clinical and biopathological characteristics.

	Neoadjuvant Therapy N = 177	Upfront Surgery N = 1099	OR (95% CI)	*p*-Value
**No. of patients (%)**	177 (13.9)	1099 (86.1)		
**Median age** (range), year *	50.4 (22.8–88.8)	62.4 (24.9–94.6)	0.54 (0.47–0.62)	<0.001
*Missing*	*2*	*2*		
**Menopausal status**				<0.001
Postmenopausal	80 (45.5)	781 (71.3)	reference	
Premenopausal	96 (54.5)	315 (28.7)	2.98 (2.15–4.11)	
*Missing*	*1*	*3*		
**Performance status (ECOG)**				0.161
0	162 (93.1)	964 (89.3)	reference	
1	11 (6.3)	75 (7.0)	0.87 (0.45–1.68)	
≥2	1 (0.6)	40 (3.7)	0.15 (0.02–1.09)	
*Missing*	*3*	*20*		
**Clinical tumor size**				<0.001
cT1	23 (13)	826 (75.2)	reference	
cT2	109 (61.6)	241 (21.9)	17.0 (10.5–27.4)	
cT3	28 (15.8)	23 (2.1)	45.7 (22.8–91.6)	
cT4	17 (9.6)	9 (0.8)	70.9 (28.5–177)	
**Clinical tumor size**				<0.001
cT1	23 (13.0)	826 (75.2)	reference	
cT ≥ 2	154 (87.0)	273 (24.8)	20.3 (12.8–32.0)	
**Clinical nodal status**				<0.001
cN0	78 (44.1)	864 (78.6)	reference	
cN1	74 (41.8)	184 (16.7)	4.46 (3.12–6.36)	
cN2	16 (9.0)	25 (2.3)	7.09 (3.64–13.8)	
cN3	9 (5.1)	26 (2.4)	3.83 (1.74–8.47)	
**Clinical nodal status**				<0.001
cN0	78 (44.1)	864 (78.6)	reference	
cN ≥ 1	99 (55.9)	235 (21.4)	4.67 (3.36–6.49)	
**Clinical stage**				<0.001
I	16 (9.0)	700 (63.7)	reference	
II	112 (63.3)	331 (30.1)	14.8 (8.63–25.4)	
III	49 (27.7)	68 (6.2)	31.5 (17.0–58.4)	
**Histotype**				<0.001
Ductal	126 (74.6)	662 (60.5)	reference	
Lobular	5 (3.0)	160 (14.6)	0.16 (0.07–0.41)	
Other	38 (22.5)	273 (24.9)	0.73 (0.50–1.08)	
*Missing*	*8*	*4*		
**Grading**				<0.001
G1	5 (3.5)	233 (22.0)	reference	
G2	49 (34.8)	559 (52.8)	4.08 (1.60–10.4)	
G3	87 (61.7)	267 (25.2)	15.0 (5.99–37.6)	
**Grading**				<0.001
G1 or G2	54 (38.3)	792 (74.8)	reference	
G3	87 (61.7)	267 (25.2)	4.78 (3.31–6.90)	
*Missing*	*36*	*40*		
**Ki-67 value**				<0.001
<20	51 (37.2)	588 (59.7)	reference	
≥20	86 (62.8)	397 (40.3)	2.50 (1.73–3.61)	
*Missing*	*40*	*114*		
**Phenotypic subgroup ^§^**				<0.001
HER2 negative/HR positive	66 (37.7)	858 (79.8)	reference	
HER2 positive/HR positive	46 (26.3)	119 (11.1)	5.03 (3.29–7.67)	
HER2 positive/HR negative	33 (18.9)	29 (2.7)	14.8 (8.47–25.9)	
Triple negative	30 (17.1)	69 (6.4)	5.65 (3.44–9.29)	
*Missing*	*2*	*24*		

**Legend:** N: number of subjects, Q1–Q3: First–third quartile, Min-Max: minimum–maximum values. * Odds Ratio estimated for 10-years unit. ^§^ Hormonal receptor status cut off: 1%. HER2 status positive if: IHC 3+ or IHC 2+ and amplified (by FISH/SISH/CISH) or amplified (by FISH/SISH/ CISH). **Notes.** The treatment of four patients with stage I-II-III invasive breast cancer was missing.

**Table 2:** Multivariable logistic regression models evaluating the probability of administrating neoadjuvant systemic therapy in stage I-II-III breast cancer patients.

N = 1070	OR (95% CI)	*p*-Value
**Menopausal status**		<0.001
Post-menopausal	reference	
Pre-menopausal	3.17 (1.97–5.11)	
Clinical tumor size *		<0.001
cT0 or cT1	reference	
cT ≥ 2	20.7 (11.2–38.5)	
Clinical nodal status *		0.005
cN0	reference	
cN > 1	1.98 (1.23–3.18)	
**Grade**		0.001
G1 or G2	reference	
G3	2.55 (1.44–4.51)	
**Ki-67 value**		0.071
<20%	reference	
≥20%	0.59 (0.33–1.05)	
**Phenotypic subgroup ^§^**		<0.001
HER2 negative/HR positive	reference	
HER2 positive/HR positive	2.52 (1.32–4.79)	
HER2 positive/HR negative	5.12 (2.07–12.6)	
Triple negative	3.32 (1.58–6.99)	

* By clinical TNM; **^§^** Hormonal receptor status cut off: 1%. HER2 status positive if: IHC 3+; IHC 2+ and amplified (by FISH/SISH/CISH); amplified (by FISH/SISH/ CISH).

**Table 3:** Patients with stage I-II-III BC treated with neoadjuvant therapy according to breast cancer phenotypic subgroup.

	Stage I-II-III Patients N = 1099	Stage I-II-III Patients Treated with Neoadjuvant Therapy N = 177	%
**Phenotypic subgroup ^§^**			
HER2 positive/HR positive	165	46	27.9
HER2 positive/HR negative	62	33	53.2
HER2 negative/HR positive	924	66	7.1
Triple negative	99	30	30.3
*Missing*	*26*	*2*	

**Legend:** N: number of subjects. **^§^** Hormonal receptor status cut off: 1%. HER2 status positive if: IHC 3+ or IHC 2+ and amplified (by FISH/SISH/CISH) or amplified (by FISH/SISH/CISH). **Notes.** Number of stage I-II-III patients treated with neoadjuvant therapy is statistically associated with phenotypic subgroup (*p*-value < 0.0001).

**Table 4:** Patient with cT > 2 cm and/or cN-positive status at diagnosis treated with neoadjuvant therapy according to breast cancer phenotypic subgroup.

	No. of Patients (Stage I-II-III) with cT > 2 cm and/or cN-Positive Status at Diagnosis	No. of Patients Treated with Neoadjuvant Therapy	%
**Phenotypic subgroup ^§^**			
HER2 positive/HR positive	95	42	44.2
HER2 positive/HR negative	42	29	69.1
HER2 negative/HR positive	366	63	17.2
Triple negative	53	26	49.1
**Total**	**556**	**160**	**28.8**

**^§^** Hormonal receptor status cut off: 1%. HER2 status positive if: IHC 3+; IHC 2+ and amplified (by FISH/SISH/CISH); amplified (by FISH/SISH/CISH). **Notes.** Number of stage I-II-III patients with cT > 2 cm and/or cN-positive status at diagnosis treated with neoadjuvant therapy is statistically associated to phenotypic subgroup (*p*-value < 0.0001).

**Table 5:** Type of neoadjuvant systemic therapy according to breast cancer phenotypic subgroup.

Phenotypic Subgroup ^§^	No. of Patients Treated with Neoadjuvant Therapy	CT	CT + AntiHER2 Agent(s)	HT	HT + AntiHER2 Agent(s)
HER2 positive/HR positive	46 (100%)	5 (10.9%)	39 (84.8%)	0	2 (4.3%)
HER2 positive/HR negative	33 (100%)	2 (6.1%)	31 (93.9%)	0	0
HER2 negative/HR positive	66 (100%)	59 (89.4%)	0	7 (10.6%)	0
Triple negative	30 (100%)	30 (100%)	0	0	0
*Missing*	*2*				

**Legend.** CT: chemotherapy; HT: hormonal therapy. **^§^** Hormonal receptor status cut off: 1%. HER2 status positive if: IHC 3+ or IHC 2+ and amplified (by FISH/SISH/CISH) or amplified (by FISH/SISH/CISH). **Notes.** The type of neoadjuvant therapy is statistically associated with phenotypic subgroup (*p*-value < 0.0001).

**Table 6:** Neoadjuvant chemotherapy regimens (±anti-HER2 agent) administered in patients according to breast cancer phenotypic subgroup.

Type of Neoadjuvant CT ± Anti-HER2 Agent	HER2+/HR+ N (%)	HER2+/HR− N (%)	HER2−/HR+ N (%)	Triple Negative N (%)
Anthra-based	1 (2.2)	1 (3.0)	1 (1.7)	2 (6.7)
Taxane-based	1 (2.2)	0 (0.0)	0 (0.0)	0 (0.0)
Anthra and taxane-based	6 (13.3)	1 (3.0)	58 (98.3)	28 (93.3)
Anthra-based + AntiHER2 agent	0 (0.0)	1 (3.0)	0 (0.0)	0 (0.0)
Taxane-based + AntiHER2 agent	9 (20.0)	10 (30.3)	0 (0.0)	0 (0.0)
Anthra and taxane-based + AntiHER2 agent	27 (60.0)	20 (60.6)	0 (0.0)	0 (0.0)
CT no anthra or taxane based	1 (2.2)	0 (0.0)	0 (0.0)	0 (0.0)
**Patients treated with CT ± anti-HER2 agent:**				
**TOTAL**	**46 (100)**	**33 (100)**	**59 (100)**	**30 (100)**

**Notes.** Hormonal receptor status cut off: 1%. HER2 status positive if: IHC 3+ or IHC 2+ and amplified (by FISH/SISH/CISH) or amplified (by FISH/SISH/ CISH).

**Table 7:** Surgery after neoadjuvant therapy.

	Patients Treated with Neoadjuvant Systemic Therapy N = 177
**BREAST SURGERY**	
**Patients treated with breast surgery**	168/177 (94.9%)
**Patients underwent:**	
Conservative surgery	73/176 (41.5%)
Mastectomy	95/176 (53.9%)
None	8/176 (4.5%)
Missing (Lost to follow up)	1
**AXILLARY SURGERY**	
**Patients treated with axillary surgery**	166/177 (93.8%)
**Type of axillary surgery**	
Only sentinel lymph node biopsy (LNB)	87/166 (52.4%)
Only axillary lymph node dissection (ALND)	53/166 (31.9%)
ALND after SLNB	26/166 (15.6%)

**Legend:** N: number of subjects. SLNB: sentinel lymph node biopsy; ALND: axillary lymph node dissection.

**Table 8:** Pathological response according to breast cancer phenotypic subgroup ^§^.

	HER2+/HR+ N = 46	HER2+/HR− N = 33	HER2−/HR+ N = 66	Triple Negative N = 30	Overall N = 175
**Pathological response**					
Complete Response (pCR)	23 (52.3%)	23 (74.2%)	11 (17.2%)	11 (37.9%)	68 (40.5%)
Partial Response (pPR)	20 (45.5%)	7 (22.6%)	48 (75.0%)	13 (44.8%)	88 (52.4%)
Stable Disease (SD)	1 (2.3%)	1 (3.2%)	4 (6.3%)	1 (3.4%)	7 (4.2%)
Progressive Disease (PD)	0 (0.0%)	0 (0.0%)	1 (1.6)	4 (13.8%)	5 (3.0%)
*Missing*	*2*	*2*	*2*	*1*	*7*

**^§^** Hormonal receptor status cut off: 1%. HER2 status positive if: IHC 3+ or IHC 2+ and amplified (by FISH/SISH/CISH) or amplified (by FISH/SISH/ CISH). **Legend:** N: number of subjects.

**Table 9:** Adjuvant systemic therapy administered after definitive surgery according to phenotypic subgroup breast cancer and pathological response ^§^.

	HER2+/HR+ N = 44	HER2+/HR− N = 29	HER2−/HR+ N = 56	Triple Negative N = 15	Overall N = 144
**Pathological response**					
Complete Response (pCR)	23/23 (100.0%)	21/23 (91.3%)	8/11 (72.7%)	0/11 (0.0%)	52/68 (76.5%)
Partial Response (pPR)	19/20 (95.0%)	7/7 (100.0%)	43/48 (89.5%)	11/13 (84.6%)	80/88 (90.9%)
Stable Disease (SD)	1/1 (100.0%)	1/1 (100.0%)	4/4 (100.0%)	1/1 (100.0%)	7/7 (100%)
Progressive Disease (PD)	0 (0.0%)	0 (0.0%)	1/1 (100.0%)	3/4 (75.0%)	4/5 (80.0%)
*Missing*	*1*	*0*	*0*	*0*	*1*

**^§^** Hormonal receptor status cut off: 1%. HER2 status positive if: IHC 3+ or IHC 2+ and amplified (by FISH/SISH/CISH) or amplified (by FISH/SISH/ CISH).

## Data Availability

The authors can confirm that all relevant data are included in the article.
